# Glutamate Water Gates in the Ion Binding Pocket of Na^+^ Bound Na^+^, K^+^-ATPase

**DOI:** 10.1038/srep39829

**Published:** 2017-01-13

**Authors:** Minwoo Han, Wojciech Kopec, Ilia A. Solov’yov, Himanshu Khandelia

**Affiliations:** 1 MEMPHYS−Center for Biomembrane Physics, University of Southern Denmark, Campusvej 55, DK-5230 Odense M, Denmark; 2 Department of Physics, Chemistry and Pharmacy, University of Southern Denmark, Campusvej 55, 5230 Odense M, Denmark

## Abstract

The dynamically changing protonation states of the six acidic amino acid residues in the ion binding pocket of the Na^+^, K^+^ -ATPase (NKA) during the ion transport cycle are proposed to drive ion binding, release and possibly determine Na^+^ or K^+^ selectivity. We use molecular dynamics (MD) and density functional theory (DFT) simulations to determine the protonation scheme of the Na^+^ bound conformation of NKA. MD simulations of all possible protonation schemes show that the bound Na^+^ ions are most stably bound when three or four protons reside in the binding sites, and that Glu954 in site III is always protonated. Glutamic acid residues in the three binding sites act as water gates, and their deprotonation triggers water entry to the binding sites. From DFT calculations of Na^+^ binding energies, we conclude that three protons in the binding site are needed to effectively bind Na^+^ from water and four are needed to release them in the next step. Protonation of Asp926 in site III will induce Na^+^ release, and Glu327, Glu954 and Glu779 are all likely to be protonated in the Na^+^ bound occluded conformation. Our data provides key insights into the role of protons in the Na^+^ binding and release mechanism of NKA.

The Na^+^, K^+^-ATPase (NKA) is an essential adenosine triphosphate (ATP) driven pump which belongs to the P-type ATPase family, which ubiquitously exists in mammalian cell membranes^1^, functioning as an active ion transporter^1^,^2^,^3^. For the cost of one ATP, NKA transports three intracellular Na^+^ ions out of the cell and two extracellular K^+^ ions into the cell in one pump cycle. The active transport is driven against the ions’ concentration gradients and maintains electrochemical gradients across the plasma membranes of mammalian cells^4^. NKA thus regulates e.g. secondary active transporters, signal transduction and the cell volume^5^.

The basic ion exchange mechanism of NKA is commonly described by the classical ‘Post-Albers reaction cycle’ which is based on cyclic changes between the two main conformational states: high Na^+^ affinity E1 and high K^+^ affinity E2 states^6^,^7^,^8^,^9^. The phosphorylated forms: E1P and E2P occur between the two states. In the E1P state, three Na^+^ ions are bound and occluded. After the transition to E2P, Na^+^ ions are released and two K^+^ ions bind to vacant ion binding sites. Subsequently, the E2 state is formed (via autodephosphorylation), with K^+^ ions bound and occluded. Eventually, NKA returns to the E1 state and releases K^+^ ions. Then, binding of three Na^+^ occurs, and subsequent autophosphorylation leads to the formation of the E1P state, thus closing the cycle. Various approaches have been used to capture and characterize ion-binding processes. Using simulations based on the crystal structures of potassium-bound occluded E2 state^10^,^11^,^12^ together with electrophysiology, it was suggested that K^+^ ion selectivity is governed by specific protonation of the acidic residues forming the ion binding sites^13^. Earlier, protonation of Asp926 in binding site III was proposed to be key for extracellular Na^+^ ion release^14^. Evidence of occasional passive proton transport through the NKA^15^ has further confirmed that proton movement and their transfer between acidic amino acid sidechains of the protein play an important role in the pumping cycle of NKA. Protons are also actively transported and they are proposed to play an important role in the ion binding in the closely related SERCA pump^16^.

Here, we investigate the protonation state of the sodium bound E1P state using MD simulations and DFT calculations, starting from the recently published crystal structure with three bound Na^+^ ions^12^. We determine the stability of the bound Na^+^ ions for all possible protonation states of the acidic residues that form the ion binding sites. The binding sites are located in the transmembrane region (Fig. 1), and the relevant acidic residues are Glu779 and Asp808 in site I, Glu327 and Asp804 in the site II, and Glu954 and Asp926 in the site III. All residue numbering follows the sequence of the crystal structure of a Na^+^-bound NKA preceding the E1P state from pig kidney (PDB ID: 3WGU)^12^. The five residues except Glu954 were predicted to be the key residues of cooperative Na^+^ binding^12^. However, in the present study we include Glu954 to observe the effect of protonation on the putative C-terminal pathway^14^,^17^. We use DFT calculations to capture the electronic structure of each specific protonation scheme, and to calculate Na^+^ binding energies.

Our data shows that (1) either three or four protonated amino acids exist in the ion-binding sites of the E1-like conformations, (2) Glu954 is protonated in the E1P state (3) protonation of Asp926 should lead to release of the Na^+^ ion in site III and (4) the Glu residues in each binding site act as water gates, and their protonation keeps the gates closed.

This article is organized as follows. Section II describes the simulation methods. Section III presents the results of Na^+^ binding stability and the specific role of protonation of the six acidic residues based on MD data. In the last part of section III, we discuss the DFT data of the Na^+^ binding energies. Section IV discusses the results in the context of previous computational and experimental studies.

## Simulation System and Methods

### Construction of the Na^+^ Bound Na^+^, K^+^ ATPase Model System

**Figure 1:**
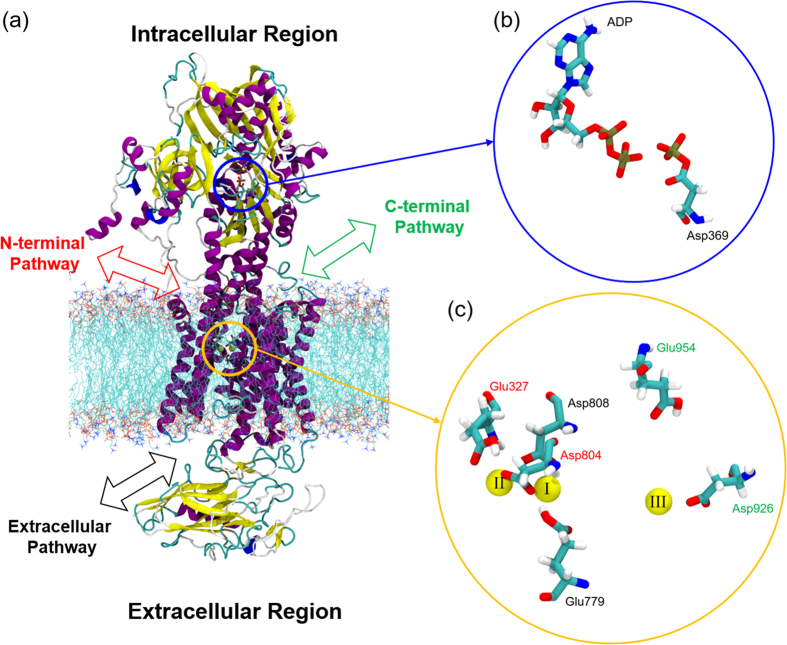
(**a**) Na^+^, K^+^-ATPase in the E1P-like state embedded in POPC lipid bilayer. For clarity, water molecules are not shown. The intracellular N- and C-terminal pathways are shown in red and green, respectively and the extracellular pathway is shown in black. Yellow and blue circles indicate the Na^+^ and ATP binding site, respectively. (**b**) The ATP binding site with bound ADP and phosphorylated Asp369. (**c**) The six key acidic residues, Glu779, Asp808, Glu327, Asp804, Glu954 and Asp926 around the binding site. The three bound Na^+^ ions are in yellow and the Roman numbers on each Na^+^ ion represent the binding sites previously defined in the crystal structure^12^.

To perform an all-atom MD simulation of an E1P like conformation of NKA, we constructed a system containing the NKA embedded in a fully hydrated 1-palmitoyl,2-oleoyl-*sn*-glycero-3-phosphocholine (POPC) membrane with Na^+^ ions (Fig. 1) based on the crystal structure (PDB ID: 3WGU)^12^. POPC has been used earlier as an effective lipid matrix to analyze ion pumps^16^,^18^. The AlF_4_- group in the crystal structure was removed and the Asp396 at the adenosine triphosphate (ATP) binding site was manually phosphorylated. An adenosine diphosphate (ADP) molecule was retained, as shown in Fig. 1(b). Na^+^, Mg^2+^ ions and water molecules from the crystal structure were retained in the model. The POPC bilayer membrane with 362 lipid molecules were equilibrated using the g_membed^19^ procedure as implemented in GROMACS version 4.6.1^20^,^21^,^22^,^23^, and hydrated with ~61,666 water molecules so the total system was consisted of ~254,000 atoms including the NKA. The system was kept electroneutral with an addition of randomly distributed 11~14 Na^+^ ions in the solution.

The Na^+^ binding pocket (Fig. 1(c)) contains six acidic residues. We generated all combinations of protonation states of the residues, resulting in 64 different systems. All other acidic residues were kept deprotonated. Table S2 in the Supporting information lists the set of all 64 protonation states, and each state is presented in this article as a symbol, *n*[*ijk*], where *n* is the number of protonated acidic residues (i.e. *n* = 0 ~ 6) and *i, j and k* in the parenthesis represent the protonation state of site I (Glu779 and Asp808), II (Glu327 and Asp804) and III (Glu954 and Asp926), respectively. We use the four symbols, 0, E, D and 2, to describe the specific protonation state of each site. ‘0’ represents no protonated acidic residues. ‘E’ and ‘D’ represent a protonated glutamic acid and protonated aspartic acid, respectively. ‘2’ represents that both Asp and Glu are protonated. For example, the symbol 3[E20] refers to a protonation state with three protonated acidic residues (*n* = 3) which are Glu779 in the site I (*i* = E), Glu954 and Asp926 in the site II (*j* = 2) but no protonated acidic residues in the site III (*k* = 0).

### Simulation details

#### MD simulations

All-atom MD simulations were performed using GROMACS version 5.0.6^20^,^21^,^22^,^23^,^24^, with the CHARMM36 force field with CMAP correction^25^,^26^,^27^,^28^ including NBFIX parameters for ions, downloaded from http://mackerell.umaryland.edu/charmm_ff.shtml. The parameters for phosphorylated aspartate were taken from a previously developed parameter set^29^. We used the TIP3P water model^25^ with Lennard-Jones interactions on hydrogen atoms. A 12 Å cutoff was used for non-bonded neighbor list, and was updated every 10 steps. The van der Waals interactions were switched off between 10 to 12 Å. Electrostatic interactions were treated with the particle mesh Ewald (PME)^30^,^31^ method. All systems were minimized with 5,000 steps using the steepest decent algorithm, followed by a 5 ns equilibration and a subsequent 50 ns production run. Three trajectories of each protonation state were run and seven additional trajectories of the stable protonation states (see later) were run for further analysis. Each sample is equilibrated with a different initial velocity distribution. Thus, we obtained total 150 to 500 ns MD trajectories for each protonation state, resulting in a total of ~12 μs of simulation. (See Table S2 in the Supporting Information for a list of all simulations). The temperature of the system was kept at 310 K with the Nose-Hoover thermostat^32^,^33^ after the equilibration run that was performed with the Berendsen thermostat^34^. The pressure was kept at 1 bar with semi-isotropic pressure coupling realized with the Parrinello-Rahman barostat^35^ after equilibration with the Berendsen barostat^34^. The Linear Constraint Solver (LINCS)^36^ algorithm was used to constrain all hydrogen containing covalent bonds. A 2 fs time step was used and trajectories were sampled every 50 ps. The data analysis was carried out using GROMACS and homemade scripts. Snapshots in figures were rendered using Visual Molecular Dynamics (VMD)^37^ and Gauss view^38^. The root mean squared fluctuation (RMSF) of the ions was calculated from:


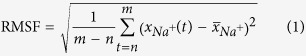


where *n* and *m* are the initial and the final simulation times used for averaging, respectively. 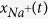 is the position of the Na^+^ ion at time *t* and 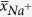 is the time-averaged position of the same ion over time (*m-n*). After calculating the RMSF of three Na^+^ ions, we averaged them out and obtained the average RMSF of three bound Na^+^ ions. We extended the three simulations of the 3[EEE] state to 250 ns, and did not find any significant changes in the values of *RMSF* and *N*_*water*_ (Supplementary Figure S2). Thus, 50 ns seems to be adequate to sample these quantities.

#### DFT calculations

**Figure 2:**
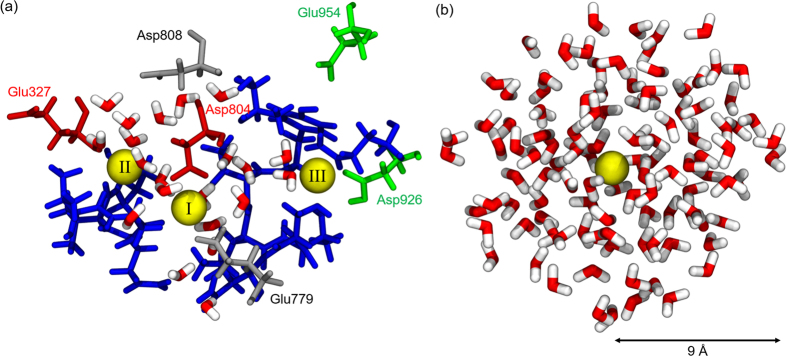
(**a**) Model system of the ion binding pocket for DFT calculations. It consists of 19 residues, three Na^+^ ions and water molecules (red and white) within a 5 Å radius of the three Na^+^ ions. Site I: Glu779 and Asp808 in grey, Site II: Glu327 and Asp804 in red, Site III: Asp926 and Glu954 in green. The other 13 residues are shown in blue: Val322, Ala323, Asn324, Val325, Pro326, Tyr771, Thr772, Leu773, Thr774, Ser775, Asn776, Thr807, and Gln923. Na^+^ ions are shown in yellow with Roman numbers that represent the binding sites. (**b**) A snapshot of single Na^+^ ion hydration shell with 9 Å radius. The hydration shells consist of ~120 water molecules which have similar number of atoms as the model system (**a**).

To calculate the binding strength of three bound Na^+^ ions, we constructed the model system of the binding sites, shown in the Fig. 2. The model contains 19 residues, three Na^+^ ions and some water molecules within 5 Å from the three Na^+^ ions. The 19 residues are Val322, Ala323, Asn324, Val325, Pro326, Glu327, Tyr771, Thr772, Leu773, Thr774, Ser775, Asn776, Glu779, Asp804, Thr807, Asp808, Gln923, Asp926, and Glu954. These are residues that contain oxygen atoms in the sidechains and the backbone, within the Na^+^ hydration radius of 2.5 Å^39^, and residues adjacent to them, including the six key acidic residues. Each calculation was performed using the atomic structure of the last snapshot of 50 ns MD trajectory. Hydrogen atoms were manually added to terminate each fragmented peptide to remove all dangling bonds. The total system size was around ~340 atoms with ~1300 electrons, similar to the other studies of reduced ion binding sites^40^,^41^.

The geometries of the systems were optimized using the DFT method with the B3LYP hybrid functional^42^,^43^ and 6–31 G** level of basis set. B3LYP was recently shown to accurately reproduce ion binding energies in metalloproteins^41^,^44^. Grimme’s dispersion correction (DFT-D)^45^,^46^ was used to model dispersion interactions. To prevent unwanted distortion in the protein backbone during optimization, we froze the positions of all 19 alpha carbon atoms. After optimization, the binding energies for each bound Na^+^ ion were obtained using the counterpoise approach, correcting for the basis set superposition error (BSSE)^47^,^48^. The Na^+^ binding energy of a single Na^+^, Δ*E*_*bind*_, is defined as


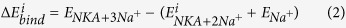


where 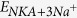 is the total energy of the system with three Na^+^ ions bound in Fig. 2(a). 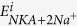 is the energy of the system without the Na^+^ ion in site *i*. 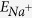 is the energy of a single Na^+^ ion with no interaction with the system (in vacuum). We calculated the Na^+^ binding energies for the three sites (*i* = I, II, and III). In addition, the hydration energy of the single Na^+^ ion in a water shell, Δ*E*_hyd_, was also calculated to compare the binding strength to specific protonation state of the model system. Note that this is different from a Na^+^ hydration free energy. Figure 2(b) shows an example of a 9 Å radius water shell with a single Na^+^ ion. A 9 Å radius water shell was selected because it consists of similar number of atoms of the binding pocket model system (~340 atoms). The water shells were chosen from 50 ns MD trajectory of single Na^+^ solvated system and 10 different snapshots with 5 ns interval were picked from the trajectory. To calculate the hydration energy, single point calculations are performed using the DFT method with the B3LYP hybrid functional and 6–31 G** level of basis set. The same protocols were used for the binding and a single Na^+^ ion hydration energy. Δ*E*_hyd_ is defined as


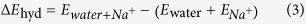


where 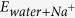 is the total energy of the system with single Na^+^ ions and the hydration shell. *E*_water_ is the energy of the hydration shell. All DFT calculations were performed using Gaussian 09 Rev. D^49^.

We did not use the QM/MM approach, which is indeed a powerful tool to study QM effects in proteins, and has its own limitations, such as limited sampling time and selection of QM/MM boundaries. Studies of isolated binding/active sites are equally interesting and informative. See for example Ngo *et al*.^41^, where energetics in isolated ion binding sites was used to benchmark current biomolecular force fields, or Dudev & Lim^50^, where a similar approach successfully identified factors governing ion selectivity between similar cations. The possibility of an alteration of the protonation scheme upon the binding of a Na^+^ ion, such as the one observed in Apell *et al*.^51^, can unfortunately not be directly simulated using our combined quantum cluster and MD approach, although the transfer of a proton within the binding site with bound Na^+^ ions is observable using our DFT method. However, we did not observe any such event in the simulations.

## Results

### Evaluating Na^+^ Bound Stability in the E1P Conformation

For determining the protonation scheme with the most stable Na^+^ binding, we performed simulations of the 64 different protonated structures that cover all the possible protonation states amongst the six acidic residues around the Na^+^ binding pocket. Our stability criterion is the following: we deemed those protonation states to be the most stable where the ions are least hydrated and the root mean square fluctuation (RMSF) of the bound ions is the lowest.

Since too many water molecules inside the binding pocket can cause the hydration and destabilization of Na^+^ ions^12^,^52^, we consider a state with fewer waters around the bound Na^+^ ions as a more stable one. Note that at least two water molecules accompany Na^+^ in all simulations.

**Figure 3:**
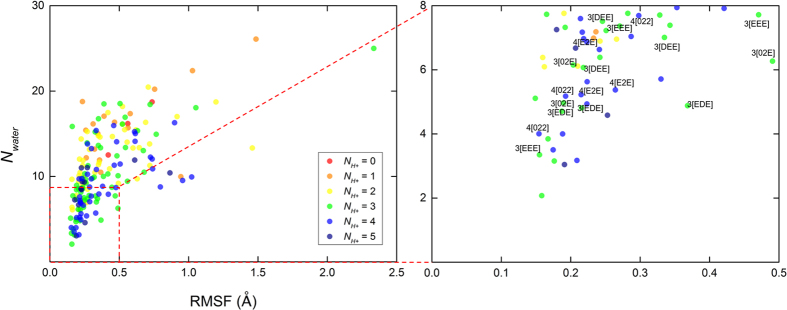
The relationship between the average number of water molecule (*N*_*water*_) within a 5 Å from the three Na^+^ ions in the binding site and average root mean square fluctuation (RMSF) of those ions. We picked a 5 Å cutoff since it covers all three binding sites and entrance of water channels (see later). The red dashed box represents *N*_*water*_ < 8 and RMSF < 0.5 Å which we defined as the region of high Na^+^ stability. The data labels of the most stable six protonation states are shown with symbols. The data points are calculated during the last 10 ns of total 50 ns MD trajectory. Each color represents a different number of protonated residues (*N*_*H*+_). Three trajectories for each system are presented, except those where Na^+^ leaves the binding sites.

The average RMSF of three bound Na^+^ ions reflects the structural stability of the bound ions. Figure 3 shows the relationship between *N*_*water*_ and RMSF. In 10 cases, the Na^+^ ions left from the binding site II, and these systems are not shown. Table S3 in the Supporting Information shows the detailed protonation scheme of each state. Finally, we selected the six most favorable protonation states which satisfy *N*_*water*_ < 8 and RMSF < 0.5 Å for all three trajectories of the MD trajectories (Table 1). We left out states where only one or two trajectories showed stable binding. These are the yellow and orange dots without a label in the right panel of Fig. 3.

Note the protonation predictions for residues constituting site III. Glu954 is protonated in all six cases, and, on the other hand, Asp926 is deprotonated except in the 4[022] state. The protonation of the residues in site III has been predicted as a crucial step in pump function in Na^+^ bound conformations in previous electrophysiological and mutational studies^53^,^54^,^55^,^56^. Our result directly shows that the specific protonation scheme in site III supports Na^+^ binding in site III. Previous studies are inconclusive about the role of Asp926 and Glu954 in binding the third Na^+^ ion. Measurement of characteristic leak current with mutations in site III revealed that the Na^+^ binding affinity in Asp926 mutants are less sensitive to extracellular sodium, whereas Glu954 mutations cause less leakage^57^. Although a recent study suggests that Asp926 coordinates site III, the role of Asp926 is described as “relatively minor” in mutagenesis experiments^58^. However, our six best candidates for the most stable Na^+^ bound protonation states suggest that at least one proton should be available to each Na^+^ binding site.

Since the Asp and Glu residues on each binding site I and II are spatially very close, we were curious about the impact of the transfer of a proton from a Glu residue to an Asp residue on the same binding site. Furthermore, Glu954 sits fairly distant from the binding site, compared to the other five acidic residues. To ascertain the role of Glu954, and to assess the role of the position of protons in each binding site, we analyzed the movement of water molecules into the binding sites, when we moved one proton from the Glu residue in each site to the Asp residue on the same site.

### Analyzing Water Pathways

**Figure 4:**
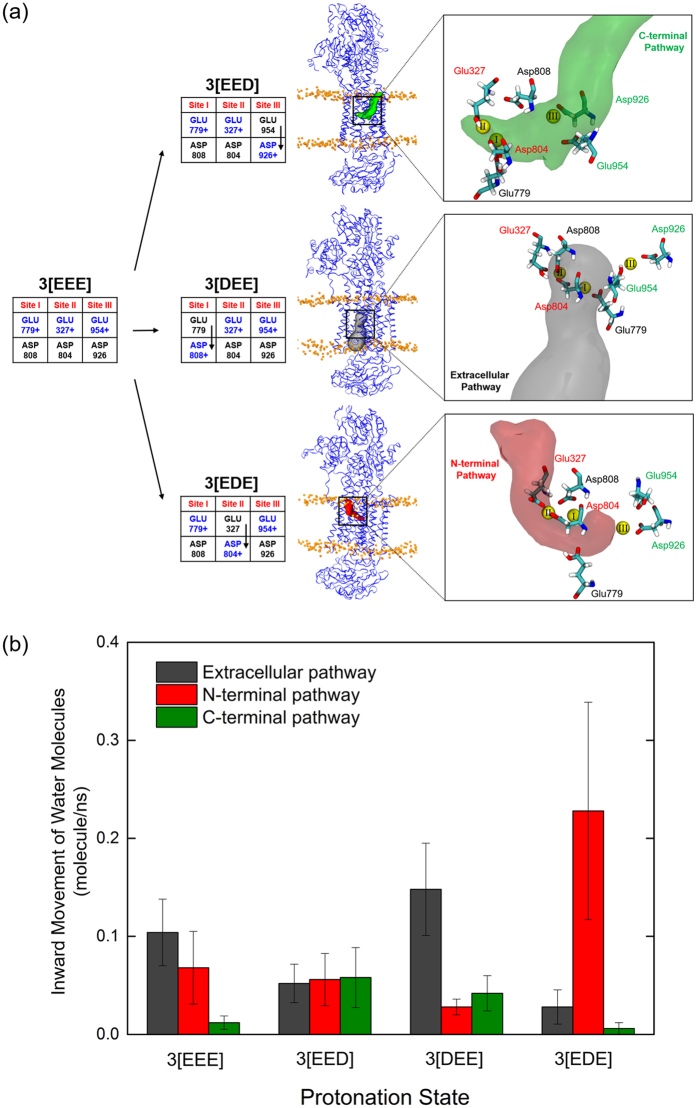
(**a**) The effect of protonation of aspartic acid on each site. The protonation schemes of 3[EEE], 3[EED], 3[DEE] and 3[EDE] states are shown. The residues in blue with a + sign are protonated. The molecular description of each protonation state is shown below. The snapshots of each water pathway were constructed using CAVER^59^ and are shown on the right. The protein structures are selected from a 50 ns MD trajectory. (**b**) The movement of water molecules into the binding site. Standard error is used for the error bars. The high error bars reflect the difference in movement of water molecules between the 10 trajectories of each simulation. Water pathways open in some instances, and remain closed in others.

The systems analyzed for the water pathways are shown in Fig. 4. In short, we started with 3[EEE], where all three of Glu327, Glu779 and Glu954 are protonated, and moved one proton from each Glu to the corresponding Asp residue on the same binding site. Seven extra simulations for each of these four systems were performed for better statistics. The water molecule movement through each pathway was calculated as described in the Supporting Information. There are three water pathways in the NKA, including the contentious C-terminal pathway^14^,^58^, and each pathway is closely related to the acidic residues around each binding site. The extracellular pathway is near site I and two intracellular pathways, N- and C-terminal pathway are near site II and site III, respectively^12^,^14^ (Fig. 4(a)). Whenever a proton was moved from a Glu to an Asp residue, the corresponding water pathway allowed entry of water. The snapshots in Fig. 4(a) show opened water pathways where the Glu residue was deprotonated. The pathways are calculated by CAVER 3.0^59^ and have the highest priority among the channels connected to the intra/extracellular region (See the Supporting Information for details). In addition, whenever a Glu residue in a specific site was deprotonated, the Na^+^ in that site moved closer to the glutamic acid and became hydrated (data not shown). The movement of water molecules through each pathway increased when the pathway was opened by Glu deprotonation (Fig. 4(b)). The large error bars reflect the difference between opened and closed pathway amongst the ten MD trajectories. The water pathways do not open in all ten trajectories.

The transition from state 3[EEE] to state 3[EED] reflects the proton transfer from Glu954 to Asp926 in site III. When Glu954 is deprotonated, the movement of water molecules in the C-terminal pathway increases and site III has the highest water accessibility (Fig. 4). Thus, the protonation state of Asp926 and Glu954 in site III controls the water accessibility of the C-terminal pathway as previously predicted^14^. In the same manner, the 3[DEE] and 3[EDE] states show an increase of the movement of water molecules in the extracellular and N-terminal pathways, which are correlated to sites I and II, respectively. In particular, the 3[EDE] state (with deprotonated Glu327) shows a large increase of movement of water molecules through site II indicating that Glu327 should be protonated to stabilize Na^+^-bound site II. We hypothesize that the glutamic acid residue in each binding site acts as a water gate when the ions are bound: if it is protonated, a water pathway is closed. On the other hand, if it is deprotonated, the water pathway opens. Interestingly, the protonation states 4[E2E] and 4[022], which have 4 protons, do not follow the same pattern as states with 3 protons. They show less water accessibility compared to the 3 protons cases though Glu327 is deprotonated in the 4[022] state (see Figure S1 in the Supporting information). An extra proton in the binding sites inhibits water circulation inside the binding pocket despite the deprotonation of the glutamic acid in the binding site.

To gain insight into Na^+^ binding affinity differences between selected stable protonation states, we used DFT calculations to optimize electronic structures and obtain the binding strength of the three bound Na^+^ ions. Such calculations include electronic effects (e.g. polarization and charge transfer), absent in classical force fields, and which were found to be crucial for an accurate description of ion-protein interactions^41^.

### Calculation of Na^+^ Binding Strength

We picked the last snapshots from each of the seven MD trajectories, and for the 3[EED] state and calculated Na^+^ binding energies of three bound Na^+^ ions. The reported binding energies are averages over three different snapshots used for initial geometry optimization. We assume that the differences in binding affinities between selected protonation states are reflected in Na^+^ binding energies between the binding pocket and the ion. For comparison, we use the binding energy of a single Na^+^ ion in a shell of water molecules. Consequently, if the binding energy of all three Na^+^ ions to the ion pocket is lower than the binding energy to the water shell, then this state is likely to be stable with all three Na^+^ ions bound. Conversely, if the binding energy to the pocket is higher than to the water shell, it renders such a state as an unstable one.

Due to the high number of charged residues (three positively charged ions and three or two negatively charged amino acid side chains), the interactions in the NKA binding pocket are dominated by electrostatics. The systems with 3 protons have an average binding energy of Na^+^ ions of about 180 kcal/mol, which compared with 170 kcal/mol obtained in a water shell suggests that these states are stable also from the energetic perspective. On the other hand, inclusion of one additional proton (that is, from states 3[*ijk*] to 4[*ijk*]) shifts the Na^+^ binding energy by ca. +30 kcal/mol, suggesting that with 4 protons bound, the binding pocket of the NKA would have significantly reduced binding affinities. Thus, it is likely that stats with 3 protons can bind a free Na^+^ ion from aqueous media (hydrated state), but states with 4 protons cannot.

Remarkably, only the binding energy of site III of the 3[EED] system is similar to the Na^+^ hydration energy. Unlike sites I and II, the protonation of Asp926 in the site III largely reduces the binding strength in the site III and this result is accordance with the model from previous mutation studies^57^. In the model, protonation of Asp926 triggers the release of Na^+^ in the site III. The 4[022] state, which has Glu954 and Asp926 protonated, also shows less Na^+^ binding energy compared to the 4[E2E] state which has Asp926 deprotonated. Clearly, protonation of Asp926 significantly reduces the binding strength of Na^+^ ion to site III.

## Discussion

We have investigated the protonation levels of the acidic residues in the binding site of the Na^+^ bound E1P conformation of Na^+^, K^+^ ATPase using MD and QM (DFT) approaches. From the MD data, we used the average number of water molecules around the three Na^+^ ions and the average RMSF of the Na^+^ ions as indicators for evaluating the stability of the bound Na^+^ ions in the E1P state. The use of the irreversible protonation model in the MD simulations gives us useful information about ‘improbable’ protonation states that show unstable Na^+^ binding. Based on this analysis, we picked six stable protonation states. These states had either 3 or 4 protons in the binding site. Interestingly, Glu954 from binding site III was protonated in all stable states. The movement of water molecules into the binding sites was regulated by (de)protonation of specific Glu and Asp residues. The hydration of a binding site increases significantly when the Glu acid residue associated with that binding site is deprotonated. Thus, Glu residues in the ion-binding pocket of NKA act as water gates and regulate water accessibility of each site. Our results are in accord with the gating function of glutamic acid in the highly related sarcoplasmic reticulum ATPase (SERCA) when it occludes bound Ca^2+^. Upon mutation of a Glu residue to Gln near the Ca^2+^ binding site II, dissociation of bound Ca^2+^ occurs rapidly^60^.

**Figure 5:**
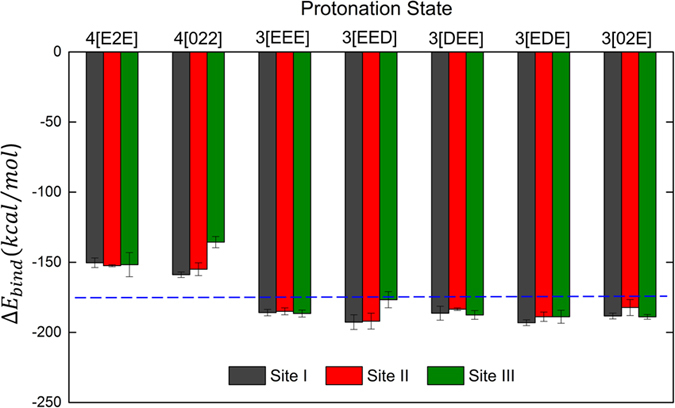
Na^+^ binding energies, Δ*E*_*bind*_ of the stable states with 3 and 4 protons. The blue dashed line represents the hydration energy of a single Na^+^ ion in a 9 Å radius water shell. The standard error for the hydration energy is ±1.39 kcal/mol. The 3[EED] state is included to observe the effect of protonation on Asp926 in site III. Each colored column represents the Na^+^ binding energy of each site. Each value is average of three replicas and standard error is used for the error bars.

Unlike the stable states with 3 protons, stable states with 4 protons, 4[E2E] and 4[022], show reduced movement of water molecule even when the Glu327 is deprotonated in 4[022]. (see Figure S1 in the Supporting information). We speculate that the state with 4 protons reduces inward movement of water molecules, and thus follows the occlusion of the E1P state. We further speculate that after such protonation, the fully closed (occluded) E1P state is formed and it is ready to transition to the E2P state, which it then ready to transition to the E2P state which is able to release one of Na^+^ ions. According to a recent study, E2P state shows significantly lower proton affinity than E1 state^61^. The reduced inward movement of water molecules may induce the lower proton affinity of E2P state. The crystal structure of the potassium-bound E2 conformation shows only few water molecules around the ion binding pocket, and in our previous simulations of this state (Kopec and Khandelia, unpublished data) the binding pocket was mostly devoid of water molecules. Therefore, we assume that a similar mechanism is at play in the E1P occluded state, and no significant number of water molecules is present at the binding sites in the occluded states of NKA. The notion that stable states with 4 protons inhibit the movement of water molecules in the ion-binding pocket provides a possible functional role of protonation during occlusion. Subsequently, such an occluded state, with 4 protons bound, should be in principle ready to release one of Na^+^ ions. Experimental data suggest that Na^+^ from the site III is the first ion to be released^57^. The Na^+^ binding energy calculations with DFT method support the above hypothesis. In Figure 5, only the 3[EED] state, which has a protonated Asp926 on site III, shows a binding strength similar to the Na^+^ hydration energy. On the other hand, the Na^+^ binding strength is the same or is higher than the hydration energy in the 3[DEE] and 3[EDE] states, where Asp residues on binding sites I and II are protonated. Thus, the protonation of Asp926 in site III has a larger influence on the Na^+^ binding strength than Asp808 in site I and Asp804 in the site II. Thus, the protonation of Asp926 seems to be involved in the induced release of the Na^+^ in site III, in line with previous reports^14^,^57^. Without such protonation in the E1P state, it is difficult to devise a mechanism in which bound Na^+^ would spontaneously leave the protein, due to its strong binding to Asp926. The other possibility is that the energy transduced into the transmembrane section of the protein from the phosphorylation reaction is itself sufficient to release Na^+^ by disrupting Na^+^ binding coordination. We envision that it is possible that the protonation and the phosphorylation are coupled each other, and together release Na^+^.

Based on Na^+^ binding energy data with 3 protons bound, we find that the most weakly bound Na^+^ ion is the one in site III when Asp926 is protonated. The protonation state, which satisfies this condition, is the 4[022]-like state. It shows low movement of water molecules and a low site III Na^+^ binding strength (compared to the Na^+^ hydration energy). To form a 4[022]-like state, the site III water channel should open first to form a water wire for transporting a proton that would bind to Asp926. Or, protons must piggyback on acidic amino acids to the center of the transmembrane regions. We do not find any such network of amino acids that can protonate Asp926. Thus, we propose that 3[EED]-like C-terminal pathway open conformation should be formed before the 4[022]-like fully occluded state.

Our DFT results also corroborate previous mutation experiments. Mutations at either Asp926 or Glu954 lower the rate of extracellular Na^+^ rebinding to a similar extent^14^. However, the Na^+^ affinity is lowered more for Asp926 mutants^14^,^54^,^62^. Furthermore, Asp926 mutants (Asp926Asn) are less sensitive to extracellular Na^+^ 55, in agreement with our data for the 3[EED] state, where we also observe a reduced Na^+^ binding affinity. Here, we assume that the Asp → Asn mutation mimics Asp protonation.

The acidic residues in the ion-binding pocket are located in the middle of the transmembrane domain. The effect of altering the protonation states of these residues can certainly have a significant impact on the conformations of the transmembrane domain, as demonstrated for the sarco/endoplasmic reticulum Ca^2+^-ATPase previously^16^,^62^,^63^. It is likely that changes in the binding affinity induced by protonation are closely coupled to membrane domain structural rearrangements, and it is the interplay between the two that drives ion release. Although we do not model direct deformations of the transmembrane domain, the rearrangements are captured in the movement of water molecules measured in Fig. 4.

Like in the NKA, we anticipate that the glutamic acids in the same position in the gastric H^+^/K^+^ pump can regulate the binding affinity of K^+^ and proton transport. Because of the limited free energy available for ATP hydrolysis, the stoichiometry of transported cations has been thought to vary from 2 H^+^/2 K^+^ to 1 H^+^/1 K^+^, depending on the decrease in luminal pH^64^,^65^,^66^. In our MD model, the protonated glutamic acid, Glu779, near the extracellular pathway will be protonated under the lower pH level of luminal region. So the model can indirectly mimic the protein conformation at lower pH. Since protonation of the glutamic acid reduces the movement of water molecules, we can speculate that protonation of Glu795 (Glu779 in the NKA) in the H^+^/K^+^ pump can reduce the proton transport ratio. Thus, the H^+^/K^+^ transport will not only depend on the stoichiometry but also on the conformational changes induced by pH, mediated by reversible protonation of Glu795. In SERCA, Ca^2+^ carry an additional charge of +2. The additional charge may allow the residues Asp926 (Asn911 in SERCA) and Glu954 (Ser940 in SERCA) to remain in a constitutively protonated state.

To the best of our knowledge, our study is the first combined MD-DFT investigation on energetics of Na^+^ binding to the E1P conformation of NKA. Our DFT calculations show that the presence of three protons in the binding pocket tunes the electrostatic interactions to be of similar magnitude to those of a single Na^+^ ion in water, which allows for robust ion binding in physiologically relevant concentrations of Na^+^. However, for a quantitative comparison with experimental dissociation constants, binding free energies would need to be calculated. Remarkably, those systems with three protonated residues are also the most structurally stable in our MD simulations. Furthermore, the shift of ~30 kcal/mol in an average Na^+^ binding energy between systems with 3 and 4 protons demonstrate how (de)protonation reactions of acidic residues help NKA change its affinity for cations, which might play a crucial role in efficient release of bound ions. We would like to remind the reader, here that the Na^+^ binding energy does not take into account entropic effects, and is thus different from free energy of ion-binding While this manuscript was in preparation, Rui et al. investigated Na^+^ and K^+^ selectivity of the binding sites by using the alchemical free energy perturbation (FEP) calculations^67^. They suggest a ‘self-correcting’ mechanism explained by the high free energy barrier toward the occlusion step when the wrong type of ion is loaded into the binding pocket. In charge-neutralizing mutations in the ion-binding pocket, they found that all the three sites remain Na^+^ selective in the partly occluded E1P state in both Asp804Asn and Asp808Asn, whilst Asp926Asn causes the sites to lose most of the Na^+^ selectivity. Thus, both the FEP calculations67 and our DFT calculations underline the importance of the role of Asp926 in the Na^+^ bound E1P state. The Post-Albers scheme suggests that transported ions are bound to the protein only transiently, and that they spontaneously leave the ion-binding pocket after major conformational changes. As suggested previously by Poulsen *et al*.^14^, we show how protonation of specific acidic residues in the binding pocket of NKA would effectively reduce its affinity for bound cations and effectively facilitate their release.

## Additional Information

**How to cite this article:** Han, M. *et al*. Glutamate Water Gates in the Ion Binding Pocket of Na^+^ Bound Na^+^, K^+^-ATPase. *Sci. Rep.* **7**, 39829; doi: 10.1038/srep39829 (2017).

**Publisher's note:** Springer Nature remains neutral with regard to jurisdictional claims in published maps and institutional affiliations.

## Supplementary Material

Supporting Information

## Figures and Tables

**Table 1:** The six selected protonation states which satisfy *N*_*water*_ < 8 and RMSF < 0.5 Å in all three trajectories.

*N*_*H*+_	Symbol	Site I		Site II		Site III	
		Glu779	Asp808	Glu327	Asp804	Asp926	Glu954
3	**3[EEE]**	+	−	+	−	−	+
	**3[DEE]**	−	+	+	−	−	+
	**3[EDE]**	+	−	−	+	−	+
	**3[02E]**	−	−	+	+	−	+
4	**4[E2E]**	+	−	+	+	−	+
	**4[022]**	−	−	+	+	+	+

Each symbol in column 2 represents a specific protonation state. The first number represents the number of protonated acidic residues, ***N***_***H**+*_, and the number in parenthesis is an indexing number of given ***N***_***H***+_. The + and − sign refer to protonated and deprotonated states, respectively.
