# Supplementary material for: Glutamate Water Gates in the Ion Binding Pocket of Na+ Bound Na+, K+-ATPase
Source: Sci Rep. 2017 Jan 13;7:39829. doi: 10.1038/srep39829 (PMC5233988; doi:10.1038/srep39829)
Supplement: Supporting Information [file srep39829-s1.pdf]

Supporting Information

Glutamate Water Gates in the Ion Binding Pocket of Na^+ Bound Na^+, K^+-ATPase

Minwoo Han[1], Wojciech Kopec[1a], Ilia A. Solov'yov[2] and Himanshu Khandelia[1]

[1]MEMPHYS–Center for Biomembrane Physics, University of Southern Denmark, Campusvej 55, DK-5230 Odense M, Denmark

[a]Current Address: Computational Biomolecular Dynamics Group, Max Planck Institute for Biophysical Chemistry, 37077 Göttingen, Germany

[2]Department of Physics, Chemistry and Pharmacy, University of Southern Denmark, Campusvej 55, 5230 Odense M, Denmark

Correspondence to hkhandel@sdu.dk

Water Pathway Analysis

Movement of water molecules – To calculate the movement of water molecules, we analyzed the trajectories of each water molecules that has ever been within 2.5 Å distance from the three bound Na^+ ions during the 50 ns trajectories. We collected all the water molecules coordinate information and calculated the distance between oxygen atom in each water molecules (O) and end of carbon in sidechain of each six acidic residues (C) in the binding pocket. We optimized the structure of Glu residue and water molecule and obtained C-O distance around 3.6 Å. So we counted the water molecules that come into the cutoff distance 4 Å from the outside of the protein (> 40 Å) and checked the amino acid residue that the water molecule comes through for the first time. After counting the number of water molecules we calculate the inward and outward movement of water molecules in Figure S1.

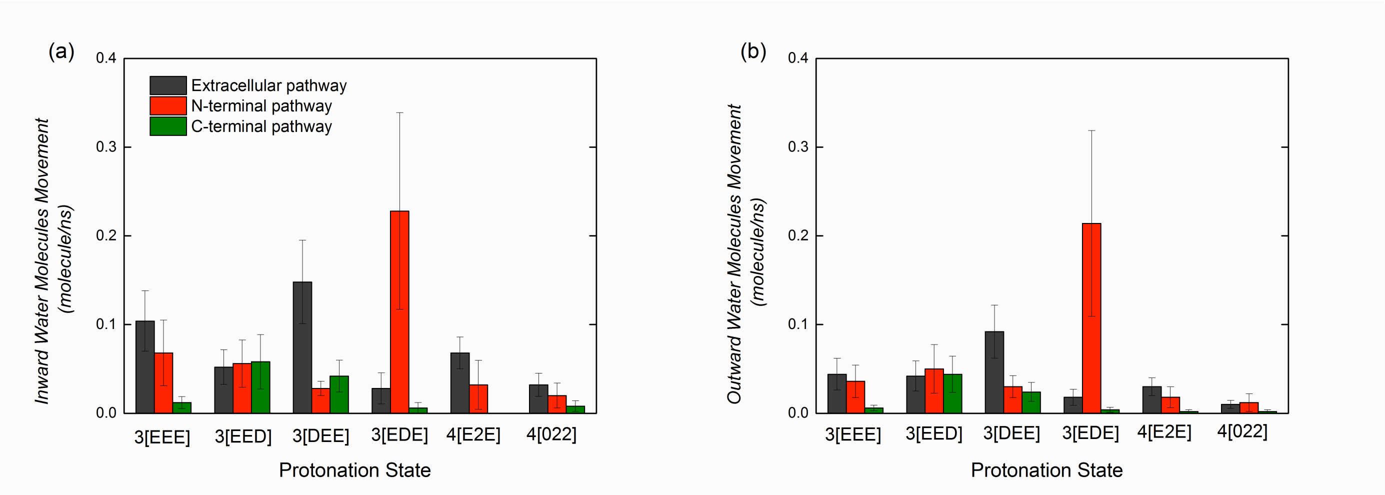

Figure S1. (a) and (b) shows the movement of inward and outward molecular water respectively. Standard error used for the error bars.

CAVER 3.0 – To demonstrate the individual water pathway of the NKA, we used the software tool, CAVER 3.0. CAVER is a software package widely used for the identification and characterization of transport pathways in static macromolecular structures. We separated the individual snapshots of the NKA structure from the MD trajectories and identified every pathway. We used a 0.9 Å probe radius, 3.5 clustering threshold and the other parameters were set as default. The starting residues were set as the six acidic residues. We obtained around 200 channels from each trajectories. Among the channels, we picked the representative water pathways, which have the highest priority among the channels, connected to intra/extracellular region. Table S1 shows the summary of the picked channels in Figure 4(a). The maximum bottleneck radius is the narrowest part of the pathway and priority is calculated by averaging the sum of channel throughputs over all snapshots—if the channel is not found in a given snapshot, a value of zero is used. Interestingly, those channels do not show high priority during 50 ns trajectories. It means the water pathways are opened temporarily during the trajectories. The average tunnel length shows the average distance of the water pathways from the intra/extracellular region to the ion binding pocket.

	Maximum bottleneck radius (Å)	Average tunnel length (Å)	Priority
**3[EED]**	0.99	31.749	0.01142
**3[DEE]**	1.08	30.352	0.01131
**3[EDE]**	1.10	27.237	0.01429

Table S1. Maximum bottleneck radius, average tunnel length and priority of picked channel calculated with CAVER 3.0

List of the 64 possible Protonation States for which simulation were run

		Site I		Site II		Site III		
N_{H+}	Symbol	Glu779	Asp808	Glu327	Asp804	Asp926	Glu954	No. copies
0	**0[000]**	-	-	-	-	-	-	3
1	**1[E00]**	+	-	-	-	-	-	3
	**1[D00]**	-	+	-	-	-	-	3
	**1[0E0]**	-	-	+	-	-	-	3
	**1[0D0]**	-	-	-	+	-	-	3
	**1[00D]**	-	-	-	-	+	-	3
	**1[00E]**	-	-	-	-	-	+	3
2	**2[0EE]**	-	-	+	-	-	+	3
	**2[0DE]**	-	-	-	+	-	+	3
	**2[0DD]**	-	-	-	+	+	-	3
	**2[0ED]**	-	-	+	-	+	-	3
	**2[002]**	-	-	-	-	+	+	3
	**2[E0E]**	+	-	-	-	-	+	3
	**2[D0E]**	-	+	-	-	-	+	3
	**2[D0D]**	-	+	-	-	+	-	3
	**2[E0D]**	+	-	-	-	+	-	3
	**2[200]**	+	+	-	-	-	-	3
	**2[EE0]**	+	-	+	-	-	-	3
	**2[DE0]**	-	+	+	-	-	-	3
	**2[DD0]**	-	+	-	+	-	-	3
	**2[ED0]**	+	-	-	+	-	-	3
	**2[020]**	-	-	+	+	-	-	3
3	**3[EEE]**	+	-	+	-	-	+	10
	**3[2E0]**	+	+	+	-	-	-	3
	**3[20D]**	+	+	-	-	+	-	3

	**3[20E]**	+	+	-	-	-	+	3
	**3[E02]**	+	-	-	-	+	+	3
	**3[D02]**	-	+	-	-	+	+	3
	**3[0E2]**	-	-	+	-	+	+	3
	**3[EED]**	+	-	+	-	+	-	3
	**3[DED]**	-	+	+	-	+	-	3
	**3[DEE]**	-	+	+	-	-	+	10
	**3[EDE]**	+	-	-	+	-	+	10
	**3[2D0]**	+	+	-	+	-	-	3
	**3[E20]**	+	-	+	+	-	-	3
	**3[EDD]**	+	-	-	+	+	-	3
	**3[DDE]**	-	+	-	+	-	+	3
	**3[02E]**	-	-	+	+	-	+	10
	**3[0D2]**	-	-	-	+	+	+	3
	**3[D20]**	-	+	+	+	-	-	3
	**3[DDD]**	-	+	-	+	+	-	3
	**3[02D]**	-	-	+	+	+	-	3
4	**4[2EE]**	+	+	+	-	-	+	3
	**4[2DE]**	+	+	-	+	-	+	3
	**4[2DD]**	+	+	-	+	+	-	3
	**4[2ED]**	+	+	+	-	+	-	3
	**4[202]**	+	+	-	-	+	+	3
	**4[E2E]**	+	-	+	+	-	+	10
	**4[D2E]**	-	+	+	+	-	+	3
	**4[D2D]**	-	+	+	+	+	-	3
	**4[E2D]**	+	-	+	+	+	-	3
	**4[220]**	+	+	+	+	-	-	3
	**4[EE2]**	+	-	+	-	+	+	3
	**4[DE2]**	-	+	+	-	+	+	3
	**4[DD2]**	-	+	-	+	+	+	3
	**4[ED2]**	+	-	-	+	+	+	3
	**4[022]**	-	-	+	+	+	+	10
5	**5[D22]**	-	+	+	+	+	+	3
	**5[E22]**	+	-	+	+	+	+	3

	**5[2D2]**	+	+	-	+	+	+	3
	**5[2E2]**	+	+	+	-	+	+	3
	**5[22E]**	+	+	+	+	-	+	3
	**5[22D]**	+	+	+	+	+	-	3
6	**6[222]**	+	+	+	+	+	+	3

Table S2. Index of possible 64 protonation states from combination of six acidic residues. Each simulation is 50 ns long. Thus, each protonation state is simulated for 150 ns, except the stable ones, which are simulated for 500 ns (10 copies)

		**Site I**		**Site II**		**Site III**	
N_{H+}	Symbol	Glu779	Asp808	Glu327	Asp804	Asp926	Glu954
4	**4[2DD]**	+	+	-	+	+	-
5	**5[E22]**	+	-	+	+	+	+
	**5[2D2]**	+	+	-	+	+	+
	**5[22E]**	+	+	+	+	-	+
	**5[22D]**	+	+	+	+	+	-
6	**6[222]**	+	+	+	+	+	+

Table S3. Na^+ leaving protonation states. At least one trajectory of those protonation states shows release of Na^+ in site II. Each symbol represents specific protonation state. + and – sign refer protonated and deprotonated residue, respectively.

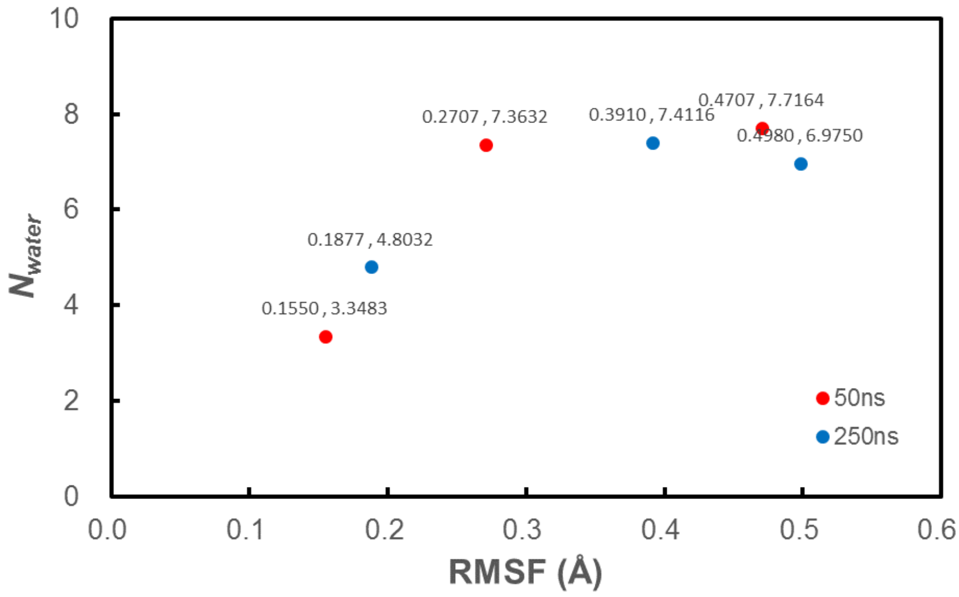

Figure S2. The relationship between the average number of water molecule (N_{water}) within 5 Å from the three Na^+ ions in the binding site and average root mean square fluctuation (RMSF) of those ions. Red and blue dots represent three sets of 50ns and 250ns simulations, respectively. The data labels show the value of RMSF and N_{water} of each point. Three MD trajectories for each system are presented.
